# Subcellular localization of HMGB1 in colorectal cancer impacts on tumor grade and survival prognosis

**DOI:** 10.1038/s41598-020-75783-2

**Published:** 2020-10-29

**Authors:** Chao-Qun Wang, Bi-Fei Huang, Yan Wang, Chih-Hsin Tang, Hong-Chuan Jin, Feng Shao, Jun-Kang Shao, Qian Wang, Yue Zeng

**Affiliations:** 1 Department of Pathology, Affiliated Dongyang Hospital of Wenzhou Medical University, 60 Wu Ning Xi Road, Dongyang, 322100 Zhejiang People’s Republic of China; 2 Department of Medical Oncology, Affiliated Dongyang Hospital of Wenzhou Medical University, Dongyang, Zhejiang People’s Republic of China; 3 Graduate Institute of Basic Medical Science, China Medical University, Taichung, Taiwan; 4 Department of Pharmacology, School of Medicine, China Medical University, Taichung, Taiwan; 5 Department of Biotechnology, College of Health Science, Asia University, Taichung, Taiwan; 6 Laboratory of Cancer Biology, Key Laboratory of Biotherapy in Zhejiang Province, Sir Run Run Shaw Hospital, Medical School of Zhejiang University, Hangzhou, Zhejiang People’s Republic of China; 7 Department of Anus and Intestine Surgery, Affiliated Dongyang Hospital of Wenzhou Medical University, Dongyang, Zhejiang People’s Republic of China

**Keywords:** Biomarkers, Oncology

## Abstract

The high-mobility group box-1 (HMGB1) protein is implicated in the development of various cancers and their proliferation. According to its function, HMGB1 shuttles between the cell nucleus and cytoplasm, assisting with nucleosome stabilization and gene transcription, or localizing in the cell membrane for outgrowth. The clinicopathologic and prognostic significance of these different subcellular locations and their correlation has been unclear in colorectal cancer (CRC). We found significantly higher rates of nuclear HMGB1 expression in CRC and colorectal adenoma tissue samples (84.0% and 92.6%, respectively) than in normal colorectal tissue (15.0%) and a significantly higher rate of positive cytoplasmic HMGB1 expression in CRC tissue (25.2%) compared with colorectal adenoma (11.8%) and normal colorectal tissue (0.0%). Positive cytoplasmic HMGB1 expression was associated with high-grade CRC, a poor prognosis, and was negatively correlated with strongly positive nuclear HMGB1 expression in CRC tissue specimens (*r* = – 0.377, *P* = 0.000). CRC patients with strongly positive nuclear HMGB1 expression had a better survival prognosis than other CRC patients. Preventing nuclear plasma translocation of HMGB1 may be a new strategy for CRC management.

## Introduction

Colorectal cancer (CRC) is one of the most common types of cancers globally, and is ranked amongst the top three malignancies in terms of morbidity and mortality^1,2^. High-mobility group box-1 (HMGB1) protein, also known as amphoterin or HMG1, was discovered in calf thymus and named according to its high electrophoretic mobility in polyacrylamide gels^3^. The biological function of HMGB1 depends on its subcellular localization and expression^4^, whereby HMGB1 can play a paradoxical role in promoting or suppressing cancer during the progression of malignant tumors^3,5–10^. Inside the nucleus, HMGB1 is a highly conserved chromosomal protein engaged in DNA repair, transcription and genome stability^3,5^. Post-translational modifications, such as phosphorylation, acetylation and methylation, enables HMGB1 to be translocated into the cytoplasm and actively released outside the cell by means of secretory lysosomes or organelles, where HMGB1 promotes inflammation, immunity, cell proliferation, autophagy, apoptosis and metastasis^3,5^.

The function and molecular mechanisms of HMGB1 remain unclear in cancer. In CRC pathogenesis, the role of HMGB1 depends on the redox status of the protein^11^. While some studies have detected the expression of HMGB1 protein in CRC^12–15^, research on the subcellular localization of HMGB1 in CRC is only available from individual reports, both of which involve only small samples of CRC tissue^16,17^. Also, the research has failed to clarify any association between different subcellular locations of HMGB1 and their clinicopathologic prognostic significance.

We performed an immunohistochemical (IHC) analysis of HMGB1 expression in CRC and colorectal adenoma tissue samples obtained from a cohort of Chinese patients. We examined the expression of HMGB1 in different subcellular locations (nucleus and cytoplasm) and analyzed the association between them. This study aimed to determine the clinicopathologic and prognostic significance of different subcellular locations of HMGB1.

## Results

### Subcellular localization and HMGB1 expression in colorectal tissue

**Figure 1:**
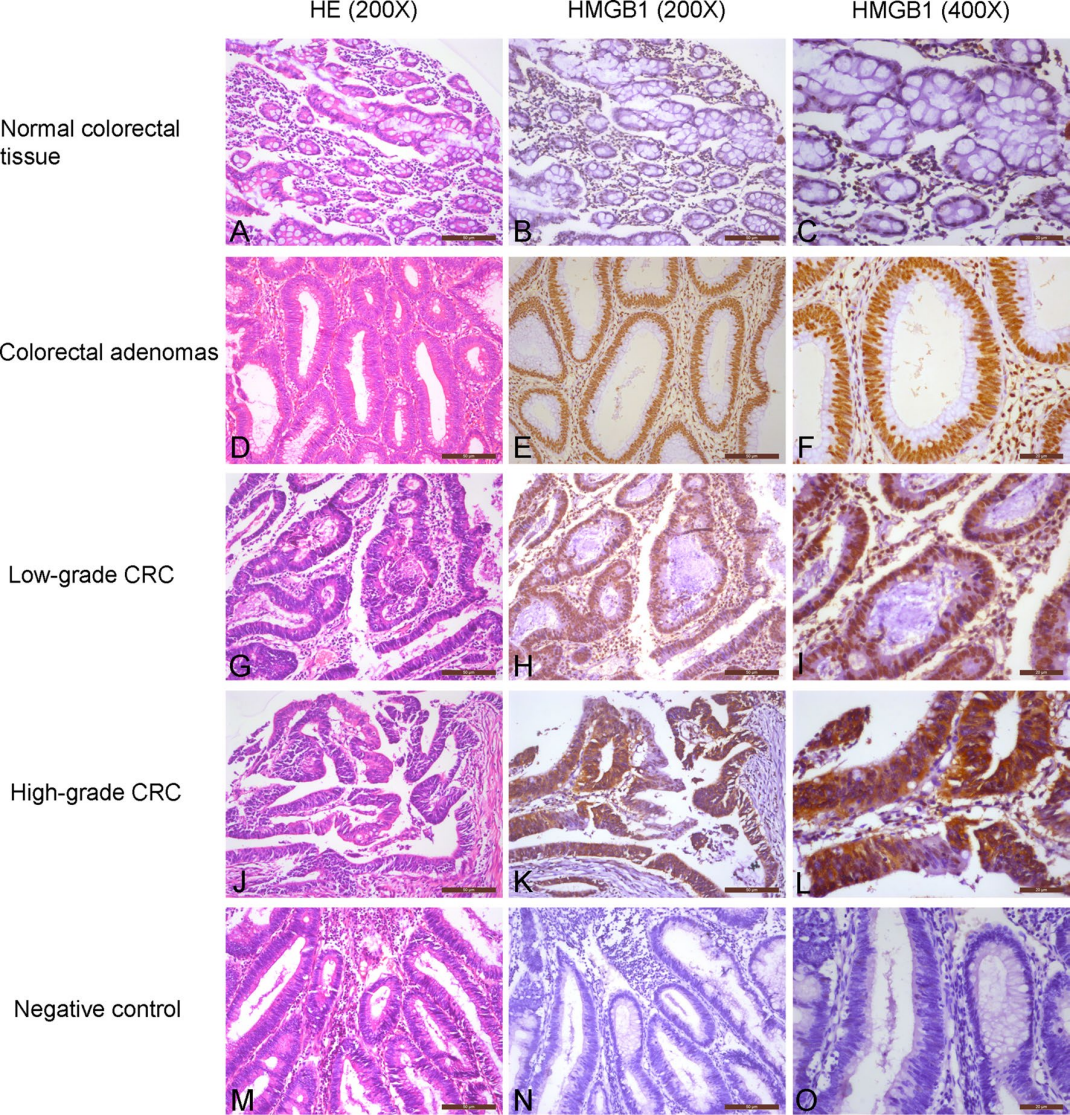
Immunochemical analysis of HMGB1 expression and subcellular localization in colorectal tissue. **(A–C)** Normal colorectal tissue, not strongly nuclear positive/cytoplasmic negative. **(D–F)** Colorectal adenomas, strongly nuclear positive/cytoplasmic negative. **(G–I)** Low-grade CRC, strongly nuclear positive/cytoplasmic negative. **(J–L)** High-grade CRC, not strongly nuclear positive/cytoplasmic positive. **(M–O)** Negative control, nuclear negative/cytoplasmic negative.

In normal colorectal tissue, HMGB1 protein was localized in the nucleus, while some cytoplasmic localization was observed in CRC and colorectal adenoma tissue samples (Fig. 1). Nuclear HMGB1 expression was generally positive in normal colorectal, adenoma and CRC tissue samples, with rates of 95.0% (19/20), 98.5% (67/68) and 99.5% (367/369), respectively. Strongly positive nuclear HMGB1 rates in normal colorectal, adenoma and CRC tissue samples were 15.0% (3/20), 92.6% (63/68) and 84.0% (310/369), respectively (*P* < 0.01, Table 1). Rates of positive cytoplasmic HMGB1 expression were 11.8% (8/68) in colorectal adenoma tissues and 25.2% (93/369) in CRC tissues (*P* < 0.01, Table 2).

**Table 1:** Strongly positive nuclear HMGB1 expression in colorectal tissue specimens.

Group	No	Nuclear HMGB1 expression	
		Not strongly positive, n (%)	Strongly positive, n (%)
Normal colorectal	20	17 (85.0%)	3 (15.0%)
Colorectal adenomas	68	5 (7.4%)	63 (92.6%)
Colorectal cancer	369	59 (16.0%)	310 (84.0%)*

* *P* < 0.01.

**Table 2:** Cytoplasmic-positive HMGB1 expression in colorectal tissue specimens.

Group	No	Cytoplasmic HMGB1 expression	
		Negative, n (%)	Positive, n (%)
Normal colorectal	20	20 (100.0%)	0 (0.0%)
Colorectal adenomas	68	60 (88.2%)	8 (11.8%)
Colorectal cancer	369	276 (74.8%)	93 (25.2%)*

* *P* < 0.01.

### The relationship between nuclear and cytoplasmic HMGB1 expression in CRC

According to levels of HMGB1 expression in the nucleus and cytoplasm, the 369 CRC patients were divided into four groups: nuclear HMGB1 expression was not strongly positive and cytoplasmic HMGB1 expression was negative in 22 patients; 254 exhibited strongly positive nuclear/cytoplasmic-negative HMGB1 expression; 37 were not strongly positive for nuclear HMGB1 expression and were cytoplasmic-positive; and 56 exhibited strongly positive nuclear/cytoplasmic-positive HMGB1 expression. Among those with cytoplasmic-positive CRC, the rate of strongly positive nuclear HMGB1 expression was 60.2% (56/93), which was significantly lower than that in the cytoplasmic-negative CRC cohort (92.0%, 254/276) (*P* < 0.01, Table 3). Spearman correlation analysis revealed a significantly negative correlation between cytoplasmic HMGB1 and nuclear HMGB1 expression in CRC tissue specimens (*r* = – 0.377, *P* = 0.000).

**Table 3:** Relationship between nuclear and cytoplasmic HMGB1 expression.

Group	No	Nuclear HMGB1 expression	
		Not strongly positive, n (%)	Strongly positive, n (%)
Cytoplasmic-negative	276	22 (8.0%)	254 (92.0%)
Cytoplasmic-positive	93	37 (39.8%)	56 (60.2%)*

* *P* < 0.01.

### The relationship between nuclear and cytoplasmic HMGB1 expression and clinicopathologic characteristics of CRC patients

As shown in Table 4, positive cytoplasmic HMGB1 expression was associated with a poor tumor grade in the CRC cohort (*P* < 0.05). CRC patients aged ≥ 60 years (27.7%, 73/264) had a higher positive cytoplasmic HMGB1 rate than patients aged < 60 years (19.0%, 20/105), but the between-group difference was not significant (*P* = 0.086). No correlation was found between strongly positive nuclear HMGB1 expression and clinicopathologic characteristics of CRC patients.

**Table 4:** Association of nuclear and cytoplasmic HMGB1 expression with clinical pathologic parameters in patients with colorectal cancer.

Variables	No	Strongly positive nuclear HMGB1 expression, n (%)	*P*-value	Positive cytoplasmic HMGB1 expression, n (%)	*P*-value
**Gender**					
Male	217	181 (83.4%)	0.707	49 (22.6%)	0.166
Female	152	129 (84.9%)		44 (28.9%)	
**Age (years)**					
< 60	105	93 (88.6%)	0.132	20 (19.0%)	0.086
≥ 60	264	217 (82.2%)		73 (27.7%)	
**Tumor site**					
Right colon	92	82 (89.1%)	0.289	25 (27.2%)	0.682
Left colon	84	70 (83.3%)		23 (27.4%)	
Rectum	193	158 (81.9%)		45 (23.3%)	
**Tumor grade**					
Low	35	29 (82.9%)	0.845	3 (8.6%)	0.017
High	334	281 (84.1%)		90 (26.9%)	
**Depth of invasion**					
Tis + T1	11	9 (81.8%)	0.748	2 (18.2%)	0.833
T2	55	45 (81.8%)		15 (27.3%)	
T3	89	78 (87.6%)		20 (22.5%)	
T4	214	178 (83.2%)		56 (26.2%)	
**Lymph node metastases**					
−	203	173 (85.2%)	0.483	47 (23.2%)	0.316
+	166	137 (82.5%)		46 (27.7%)	
**Tumor stage**					
I	47	40 (85.1%)	0.634	12 (25.5%)	0.847
II	149	127 (85.2%)		34 (22.8%)	
III	144	117 (81.3%)		39 (27.1%)	
IV	29	26 (89.7%)		8 (27.6%)	

### High levels of nuclear or cytoplasmic HMGB1 expression predict opposite effects on survival in CRC

**Figure 2:**
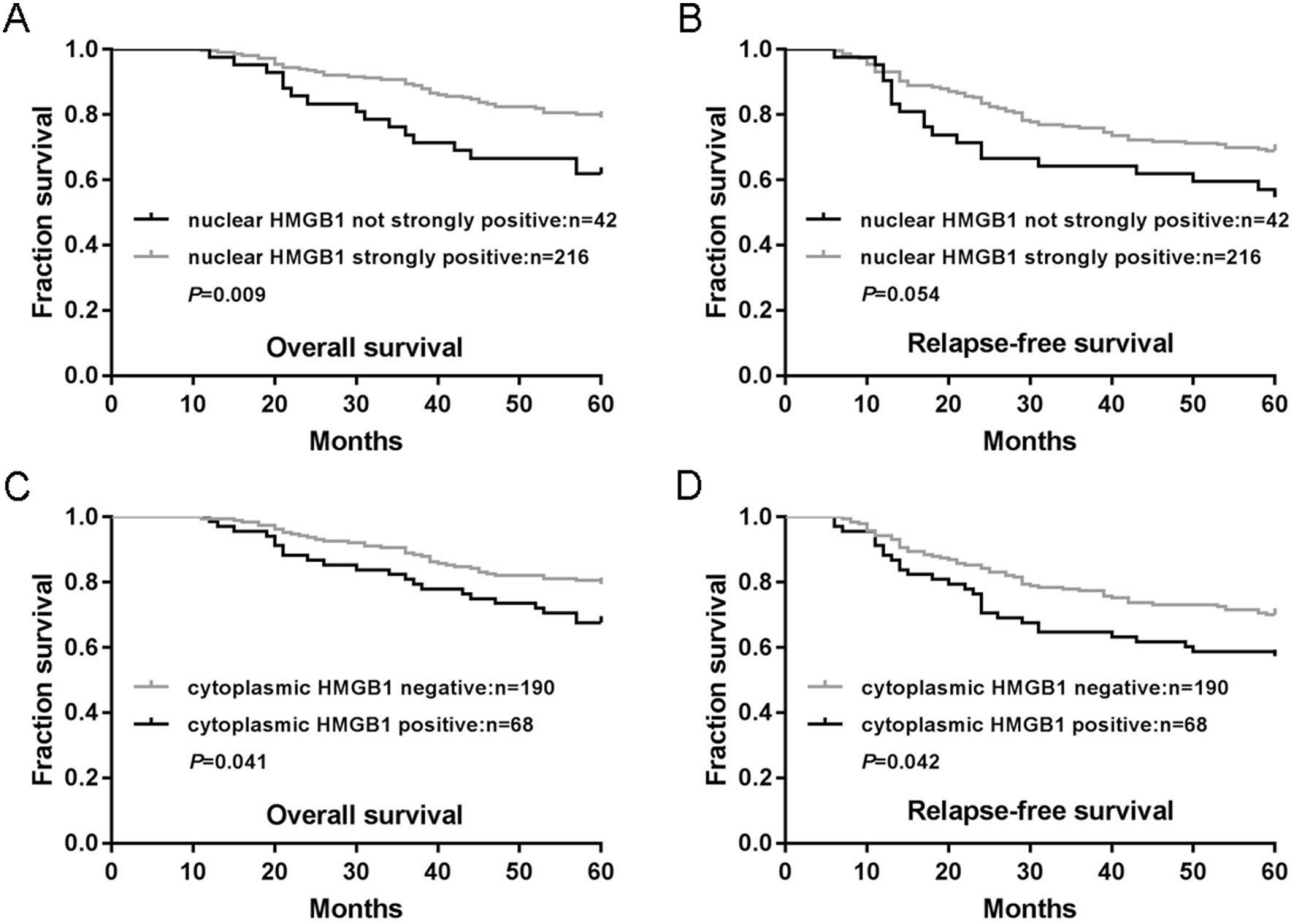
Associations between nuclear and cytoplasmic HMGB1 expression with survival of patients with colorectal cancer. **(A,B)**. Associations of nuclear HMGB1 expression with overall survival (OS) **(A)** and relapse-free survival (RFS) **(B)** were analyzed in the colorectal cancer cohort. **(C,D)**. Associations of cytoplasmic HMGB1 expression with OS **(C)** and RFS **(D)** were analyzed in the colorectal cancer cohort. *P*-values were calculated using the Mantel-Cox log-rank test.

We analyzed levels of nuclear or cytoplasmic HMGB1 expression in relation to RFS and OS rates in patients with CRC. As shown in Fig. 2A, the 216 patients whose primary tumors exhibited strongly positive nuclear HMGB1 expression had a mean OS of 54.7 months (an estimated 5-year OS rate of 79.2%), while the 42 patients whose tumors did not exhibit strongly positive nuclear HMGB1 expression had a mean OS of 49.1 months (an estimated 5-year OS rate of 61.9%, *P* = 0.009). Similarly, CRC patients with strongly positive nuclear HMGB1 expression had a better RFS than those whose nuclear HMGB1 expression was not strongly positive, although the data did not support an unequivocal association (Fig. 2B, P = 0.054).

The 68 patients whose primary tumors exhibited positive cytoplasmic HMGB1 expression had a mean OS of 51.2 months (an estimated 5-year OS rate of 67.6%), while the 190 patients whose tumors were negative for cytoplasmic HMGB1 expression had a mean OS of 54.7 months (an estimated 5-year OS rate of 79.5%, Fig. 2C, P = 0.041). Similarly, patients with cytoplasmic-positive HMGB1 expression experienced worse RFS. Patients whose primary tumors were HMGB1 cytoplasmic-positive had a mean RFS of 44.3 months (an estimated 5-year RFS rate of 57.4%), while patients whose tumors were HMGB1 cytoplasmic-negative had a mean RFS of 49.8 months (an estimated 5-year RFS rate of 70.0%, Fig. 2D, P = 0.042).

## Discussion

Scant data are available in regard to the subcellular localization of HMGB1 in malignant tumors. Kostova and colleagues found that HMGB1 subcellular localization was associated with histologic differentiation of malignant tumors, as moderately differentiated carcinomas exhibit perinuclear localization of the protein, while poorly differentiated carcinomas show a tendency for non-specific nuclear localization^18^. Data about the subcellular localization of HMGB1 in CRC is only available from two individual reports, both of which involve small samples of CRC tissue and neither has clarified the correlation between different subcellular localization sites of HMGB1 and their clinicopathologic prognostic significance^16,17^.

Our study found that in normal colorectal tissue, colorectal adenoma and CRC samples, HMGB1 is generally positively expressed in the nucleus. In colorectal adenoma and CRC tissues, the rates of strongly positive nuclear HMGB1 expression were significantly higher than the rate in normal colorectal tissue, while the rate was slightly higher in colorectal adenoma than in CRC tissue, suggesting that the enhanced expression of nuclear HMGB1 occurs in the early stage of colorectal neoplastic lesions. In addition, the positive rates of cytoplasmic HMGB1 in normal colorectal tissue, colorectal adenoma and CRC showed a gradually increasing trend, with significantly higher expression of positive cytoplasmic HMGB1 in high-grade versus low-grade CRC tissue, suggesting that cytoplasmic HMGB1 expression is closely related to CRC tumor grade.

We also found a significantly lower strongly positive rate of nuclear HMGB1 expression in cytoplasmic-positive CRC than in cytoplasmic-negative CRC, and a significant negative correlation between cytoplasmic and nuclear HMGB1 expression. Moreover, we observed that in CRC or adenoma cells subjected to cytoplasmic staining, nuclear staining was either weak or not apparent. Thus, we have revealed an obvious nuclear plasma translocation phenomenon of HMGB1 protein in CRC.

We found that cytoplasm-positive HMGB1 expression is related to a poor prognosis in CRC. Interestingly, nuclear-positive HMGB1 expression had an opposite outcome on survival prognosis, as CRC patients with strongly positive nuclear HMGB1 expression had a better prognosis than other patients. Previous studies involving different cancers concluded that HMGB1 plays a paradoxical role in promoting or suppressing cancer^3,5–10^. This is the first time that HMGB1 has been found to play both positive and negative roles in the same study population. We believe that the enhancement of nuclear HMGB1 expression in colorectal adenoma and CRC lays the molecular basis for nuclear plasma translocation for the further progress of tumors in later stages of the disease. As nuclear HMGB1 is involved in a wide range of effects, such as DNA repair, transcription and genome stability^3,5^, it is understandable that its high expression would be protective in regard to survival in CRC. We speculate that high levels of nuclear HMGB1 expression activate many downstream genes or pathways, such as epidermal growth factor receptor (EGFR)^19^ and β-catenin^20,21^. We found a significantly positive correlation between strongly positive levels of nuclear HMGB1 and EGFR positive or β-catenin positive expression in CRC tissue specimens (Supplementary materials, Table S1–S4, Figure S1). Further research into the molecular mechanisms of HMGB1 nuclear plasma translocation in CRC is necessary. Preventing the nuclear plasma translocation of HMGB1 may be a potentially useful strategy for CRC treatment.

## Materials and methods

### Patients and tissue samples

Tissue samples were obtained from 369 Chinese patients with CRC and 68 patients with colorectal adenoma. All patients were undergoing primary surgical treatment at the Affiliated Dongyang Hospital of Wenzhou Medical University (Dongyang, Zhejiang, China) between 2008 and 2015. Twenty cases of adjacent normal colorectal tissue specimens were obtained from the above-mentioned 20 CRC samples post-surgery. Clinicopathologic characteristics were determined for all patients based on their medical records. CRC patients with a median age of 69 years (range 24–94), and patients diagnosed with colorectal adenoma with a median age of 68 years (range 33–88). The characteristics and distribution of CRC population are shown in Table 4. Pathohistologic and clinical diagnosis was made according to the World Health Organization classification of tumours of the digestive system^22^. CRC patients were staged according to the eighth edition of the AJCC cancer staging manual^23^. Follow-up information was available for 258 patients with CRC; the median follow-up was 60 months (range, 6–75 months). Study approval was granted by the Ethics Committee of the Affiliated Dongyang Hospital of Wenzhou Medical University. Written informed consent forms were signed by all patients or their guardians. All study methods satisfied the relevant guidelines and regulations issued by the Affiliated Dongyang Hospital of Wenzhou Medical University.

### Tissue array preparation

We followed the methods described by Wang et al., 2020^24^.

### IHC analysis

IHC staining of paraffin-embedded tissue array sections was conducted using the Envision System (Dako, Glostrup, Denmark), as described previously^25,26^. Briefly, the sections were submerged in boiling sodium citrate (pH, 6.0) for 2 min in a pressure cooker. After being treated with 0.3% hydrogen peroxide for 10 min to block endogenous peroxidase, the sections were incubated with primary antibody for 1 h at room temperature. The sections were then incubated with secondary antibody (Dako) for 40 min at room temperature, followed by incubation with 3,3′-diaminobenzadine (Dako) at room temperature. The primary antibody were anti-HMGB1 rabbit monoclonal antibody (clone EPR3507, diluted at 1:1000, Abcam, Cambridge, England), anti-EGFR rabbit polyclonal antibody (clone 1005, diluted at 1:100, Santa Cruz Biotechnology, Santa Cruz, USA) and anti-β-catenin rabbit monoclonal antibody (clone D10A8; diluted at 1:400; Cell Signaling Technology, Boston, USA). The secondary antibody was Dako's HRP rabbit/mouse universal antibody (Dako, Glostrup, Denmark). The negative control was incubated with vehicle then with secondary antibody, without primary antibody. As positive control, we used the internal control in the colorectal tissue, to check for HMGB1 nuclear staining in the interstitial fibers and lymphocytes; a breast cancer tissue that is known to be EGFR-positive or β-catenin-positive serves as positive control for EGFR and β-catenin staining, respectively.

### Assessment of HMGB1 staining

The entire tissue array section was scanned and scored separately by 2 pathologists. The intensity of nucleus and cytoplasm HMGB1 staining was assessed in colorectal tissue. Staining intensity was scored on a 4-point scale from 0 (negative) to 1 (weak), 2 (medium), or 3 (strong)^27^. In both the nucleus and cytoplasm, scores of 0 were negative and 1 to 3 were positive for HMGB1. A score of 2 to 3 in the nucleus was considered to be strongly positive. High levels of expression are expressed as strongly positive nuclear HMGB1 and positive cytoplasmic HMGB1 expression.

### Patient follow-up

We followed the methods described by Wang et al., 2020^24^.

### Statistical analysis

Statistical analyses were conducted using SPSS version 19.0 (SPSS Inc, Chicago, IL, USA). Between-group differences were compared using a Pearson’s Chi-square test for qualitative variables. The correlation between nucleus and cytoplasm HMGB1 expression was assessed by Spearman’s correlation analysis. Patient RFS and OS rates were analyzed using the Kaplan–Meier method and compared using log-rank analysis. *P* < 0.05 was considered to be statistically significant.

## Supplementary information

Supplementary Information.

## Abbreviations

IHC: Immunohistochemistry
CRC: Colorectal cancer
HMGB1: High-mobility group box-1
RFS: Relapse-free survival
OS: Overall survival
