# Supplementary material for: Subcellular localization of HMGB1 in colorectal cancer impacts on tumor grade and survival prognosis
Source: Sci Rep. 2020 Oct 29;10:18587. doi: 10.1038/s41598-020-75783-2 (PMC7596050; doi:10.1038/s41598-020-75783-2)
Supplement: Supplementary file 1 — Supplementary Information. [file 41598_2020_75783_MOESM1_ESM.doc]

**Subcellular localization of HMGB1 in colorectal cancer impacts on tumor grade and survival prognosis**

Chao-Qun Wang^1*^, Bi-Fei Huang^1^, Yan Wang^2^, Chih-Hsin Tang^3–5^, Hong-Chuan Jin^6^, Feng Shao^7^, Jun-Kang Shao^1^, Qian Wang^1^ and Yue Zeng^1^

^1^Department of Pathology, Affiliated Dongyang Hospital of Wenzhou Medical University, Dongyang, Zhejiang, People’s Republic of China

^2^Department of Medical Oncology, Affiliated Dongyang Hospital of Wenzhou Medical University, Dongyang, Zhejiang, People’s Republic of China

^3^Graduate Institute of Basic Medical Science, China Medical University, Taichung, Taiwan

^4^Department of Pharmacology, School of Medicine, China Medical University, Taichung, Taiwan

^5^Department of Biotechnology, College of Health Science, Asia University, Taichung, Taiwan

^6^Laboratory of Cancer Biology, Key Laboratory of Biotherapy in Zhejiang Province, Sir Run Run Shaw Hospital, Medical School of Zhejiang University, Hangzhou, Zhejiang, People’s Republic of China

^7^Department of Anus and Intestine Surgery, Affiliated Dongyang Hospital of Wenzhou Medical University, Dongyang, Zhejiang, People’s Republic of China

**^*^Corresponding author:**

Chao-Qun Wang; Department of Pathology, Affiliated Dongyang Hospital of Wenzhou Medical University, 60 Wu Ning Xi Road, Dongyang 322100, China.

E-mail: [chaoqunw869@163.com](mailto:chaoqunw869@163.com)

**Supplementary materials**

IHC analyses detected EGFR expression in 232 CRC cases and β-catenin expression in 144 CRC cases. Invasive tumor cells with ≥10% membrane staining were considered to be positive for EGFR[^1^](#_ENREF_1). A case was considered to be β-catenin-positive if the percentage of positive invasive cancer cells (nuclear staining) was ≥1%[^2^](#_ENREF_2).

The rate of positive EGFR expression in CRC tissue specimens was 82.3% (191/232). We found a significantly higher level of EGFR expression in CRC tissue specimens from cases that were strongly positive for nuclear HMGB1 (84.4%, 168/199) compared with those that were not strongly positive (69.7%, 23/33; *P*<0.05, Table S1). Spearman correlation analysis revealed a significantly positive correlation between strongly positive levels of nuclear HMGB1 expression and EGFR positive expression in CRC tissue specimens (*r*=0.135, *P*=0.040) (FigureS1). No such correlation was observed between cytoplasmic HMGB1 and EGFR expression (Table S2).

The rate of positive β-catenin expression in CRC tissue specimens was 48.6% (70/144). We found a significantly higher level of β-catenin expression in CRC tissue specimens from cases that were strongly positive for nuclear HMGB1 (53.4%, 63/118) compared with those that were not strongly positive (26.9%, 7/26; *P*<0.05, Table S3). Spearman correlation analysis revealed a significantly positive correlation between strongly positive levels of nuclear HMGB1 expression and β-catenin positive expression in CRC tissue specimens (*r*=0.204, *P*=0.014) (FigureS1). No such correlation was observed between cytoplasmic HMGB1 and β-catenin expression (Table S4).

**Table S1. Relationship between nuclear HMGB1 and EGFR expression in patients with colorectal cancer**

|  |  | EGFR expression | |
| --- | --- | --- | --- |
| Group | No. | Negative, n (%) | Positive, n (%) |
| Nuclear- not strongly positive | 33 | 10 (30.3%) | 23 (69.7%) |
| Nuclear- strongly positive | 199 | 31 (15.6%) | 168 (84.4%)* |

* *P*<0.05.

Table S2. Relationship between cytoplasmic HMGB1 and EGFR expression in patients with colorectal cancer

|  |  | EGFR expression | |
| --- | --- | --- | --- |
| Group | No. | Negative, n (%) | Positive, n (%) |
| Cytoplasmic-negative | 170 | 30 (17.6%) | 140 (82.4%) |
| Cytoplasmic-positive | 62 | 11 (17.7%) | 51 (82.3%)* |

* *P*=0.987.

Table S3. Relationship between nuclear HMGB1 and β-catenin expression in patients with colorectal cancer

|  |  | β-catenin expression | |
| --- | --- | --- | --- |
| Group | No. | Negative, n (%) | Positive, n (%) |
| Nuclear- not strongly positive | 26 | 19 (73.1%) | 7 (26.9%) |
| Nuclear- strongly positive | 118 | 55 (46.6%) | 63 (53.4%)* |

* *P*<0.05.

Table S4. Relationship between cytoplasmic HMGB1 and β-catenin expression in patients with colorectal cancer

|  |  | β-catenin expression | |
| --- | --- | --- | --- |
| Group | No. | Negative, n (%) | Positive, n (%) |
| Cytoplasmic-negative | 101 | 48 (47.5%) | 53 (52.5%) |
| Cytoplasmic-positive | 43 | 26 (60.5%) | 17 (39.5%)* |

* *P*=0.155.


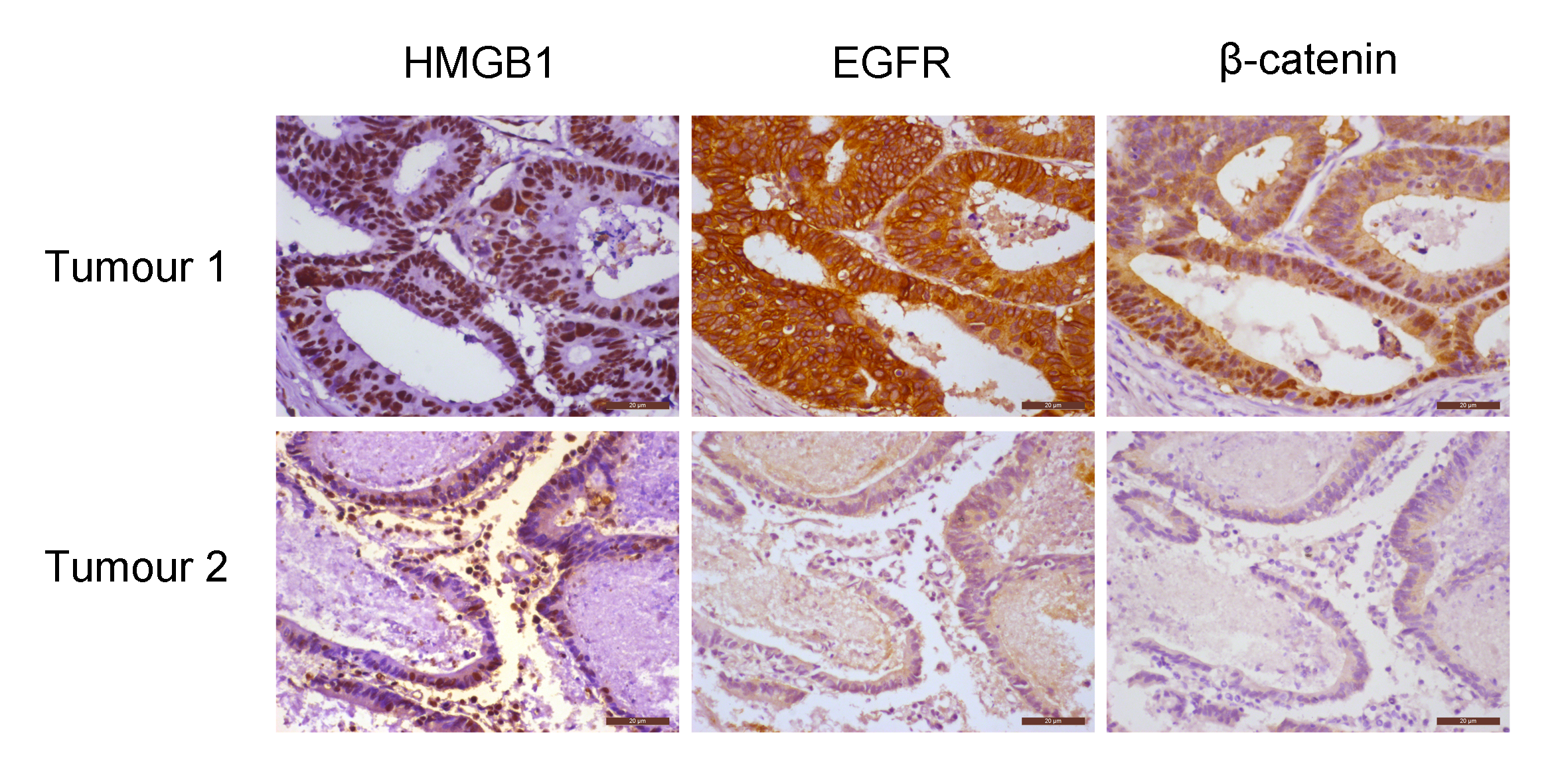


**Figure S1. A tendency of positive protein levels between HMGB1, EGFR and β-catenin in colorectal cancer**: Human colorectal cancer tissues were immune-stained with anti-HMGB1, anti-EGFR and anti-β-catenin antibodies. Representative staining pictures of tumors are shown.

**References**

1. Wang CQ, Li Y, Huang BF, Zhao YM, Yuan H, Guo D, Su CM, Hu GN, Wang Q, Long T, Wang Y, Tang CH, et al. EGFR conjunct FSCN1 as a Novel Therapeutic Strategy in Triple-Negative Breast Cancer. *Scientific reports* 2017;**7**: 15654.

2. Nguyen TH, Nguyen VH, Nguyen TL, Qiuyin C, Phung TH. Evaluations of Biomarker Status Changes between Primary and Recurrent Tumor Tissue Samples in Breast Cancer Patients. *BioMed research international* 2019;**2019**: 7391237.
